# Characteristics of FXa-storing platelets in hemophilia B mice and the influence of alcohol on the platelets

**DOI:** 10.1038/s41598-023-43649-y

**Published:** 2023-10-01

**Authors:** Wenwen Han, Rui Huang, Binbin Li, Lu Liu, Wenjue Xu, Guowei Zhang

**Affiliations:** Key Laboratory of Aging and Cancer Biology of Zhejiang Province, Department of Biochemistry and Molecular Biology, School of Basic Medical Sciences, Hangzhou Normal University, No. 2318, Yuhangtang Rd, Cangqian, Yuhang District, Hangzhou, 311121 Zhejiang China

**Keywords:** Biotechnology, Gene therapy

## Abstract

Platelet-stored activated blood coagulation factor X (FXa) has great potential in the gene therapy of hemophilia B (HB). However, we still need to understand more about the properties of FXa-storing platelets and how dietary factors affect them. We created transgenic mice called 2bFXa-HB, which had stable expression and storage of FXa in their platelets, resulting in the alleviation of the bleeding disorder in these mice. Even after inducing anti-factor IX (FIX) inhibitors in 2bFXa-HB mice, the hemorrhage phenotype could still be rescued by the expression of FXa. The activation capacity of 2bFXa-HB platelets remained unchanged, and there were no signs of elevated thrombotic risk in these mice. In an acute alcohol exposure mouse model, a single administration of alcohol reduced both the number of platelets and their activation capacity, as well as impaired coagulation function. However, it did not increase the markers of thrombotic risk in either 2bFXa-HB or HB mice. These results suggest that FXa storage in platelets is safe and effective for treatment of HB, but alcohol could impair the therapeutic effect of FXa-containing platelets.

## Introduction

Hemophilia is an X-linked recessive inheritance disease caused by a deficiency or reduction in blood coagulation factors. Hemophilia B (HB) specifically results from a deficiency in factor IX (FIX). Currently, the standard treatment for HB is replacement therapy. However, approximately 5% of treated patients develop FIX inhibitors^1^, and among those, 80% have inhibitors with more than 5 Bethesda units^2^, significantly reducing the effectiveness of FIX replacement therapy. Bypassing agents can be used to treat HB patients with inhibitors, but they have disadvantages such as a short half-life and high treatment costs^3^.

Factor X (FX) plays a pivotal role in the coagulation process. It is activated either by activated FIX (FIXa)/activated FVIII (FVIIIa) or by activated FVII (FVIIa)/tissue factor (TF), leading to the formation of activated FX (FXa). FXa, in turn, converts prothrombin into thrombin, essential for achieving hemostasis^4^.

The use of FXa supplementation in bypassing therapy offers distinct advantages in the treatment of hemophilia. FXa operates downstream of FVIII and FIX in the coagulation cascade, enabling it to trigger the coagulation process independently. FEIBA (FVIII inhibitor bypassing activity) has been employed for decades to treat hemophilia patients who have developed inhibitors, and the critical role of FXa in FEIBA's activity has been well-established^5^.

Previous research has also demonstrated the effectiveness of FXa in correcting bleeding disorders^6–8^. However, FXa's short half-life when infused into plasma^9^, coupled with a heightened risk of intravascular thrombosis^6^, presents challenges. To address these limitations, platelet-targeted gene therapy emerges as a promising solution. In our prior study^10^, we targeted the expression of FXa in platelets through lentiviral modification of hematopoietic stem cells. We confirmed that targeting FXa expression in platelets allows for the storage of FXa in platelet α-granules. This stored FXa can be released upon platelet activation at injury sites, initiating hemostasis.

Through this gene therapy strategy, we successfully alleviated the bleeding phenotype in both hemophilia A (HA) and HB mice. Importantly, this approach remains effective even in the presence of anti-FVIII or anti-FIX inhibitors. Furthermore, the presence of FXa in plasma is limited, with no signs of increased thrombotic risk observed. Therefore, platelet-targeted FXa gene therapy holds significant promise as an ideal alternative treatment for hemophilia patients, especially those who have developed anti-FVIII or anti-FIX inhibitors following replacement therapy.

In platelet-targeted gene therapy, the introduction of foreign proteins to platelets imparts new functions, making it crucial to maintain their proper functionality. Thus far, no functional alterations have been reported in platelets containing FVIII or FIX. In our previous work^10^, we demonstrated the safety of platelet-stored FXa in lipopolysaccharide-challenged HA mice.

In addition to typical prothrombotic situations, we were interested in understanding how the patients' daily life environment might affect platelet function, as this is likely the most common scenario for patients. Dietary factors play a significant role in influencing the coagulation system. Platelet function is influenced by various dietary factors, yet studies investigating whether modified platelets behave normally under different dietary conditions are still lacking.

Excessive alcohol consumption is considered a contributing factor to cardiovascular disease. Binge drinking can lead to numerous acute impairments and adverse health consequences^11,12^. Research has indicated that alcohol consumption can reduce coagulation factors, inhibit platelet aggregation, and decrease platelet counts in heavy drinkers^13^. However, other studies have suggested that acute alcohol ingestion could enhance platelet activation^14^. An experiment in a mouse model demonstrated that acute alcohol exposure reduced the expression of Bcl-XL and enhanced Bak expression, ultimately leading to platelet apoptosis through the activation of the caspase-3 pathway^15^. Therefore, it is essential to clarify the effect of alcohol on FXa-storing platelets and whether it influences the release of platelet-stored FXa."

In this study, we utilized a platelet-targeted promoter, the αIIb promoter, to drive the expression and storage of FXa in platelets. This approach resulted in the creation of a 2bFXa transgenic mouse model with an HB background, referred to as 2bFXa-HB. Our investigation aimed to assess the functionality of FXa-containing platelets, examine the therapeutic effects and safety of platelet-stored FXa in HB mice, particularly in the presence of FIX inhibitors, and evaluate the impact of acute alcohol exposure on the function of FXa-storing platelets."

## Methods and materials

### Animals

We used a *F9* knockout mouse purchased from Jackson Lab (Bar Harbor, ME, USA) as the HB mouse model. 2bFXa transgenic mice with a C57BL/6 background were constructed by Cyagen (Guangzhou, China). In the mice, the FXa cassette was constructed by replacing the activation peptide of the human FX with an Arg–Lys–Arg sequence as previously reported^10^. and the platelet-specific promoter αIIb was applied to control the expression of FXa. The αIIb promoter was from Dr. David A. Wilcox(BloodCenter of Wisconsin, WI, USA). We crossed 2bFXa mice with HB mice and screened for 2bFXa-HB transgenic mice. All mice were maintained in special pathogen-free rooms at the Animal Center of Hangzhou Normal University. All methods were approved by the Experimental Animal Ethics Committee of Hangzhou Normal University, and they were performed in accordance with the relevant guidelines and regulations. We followed the guidelines of the Animal Research: Reporting of In Vivo Experiments (ARRIVE). FIX inhibitors in 2bFXa-HB mice were developed through intraperitoneal injection of recombinant human FIX (BeneFIX, Pfizer, China) as previously described^16^. Briefly, 200 U/kg recombinant hFIX mixed with Freund’s adjuvant (SigmaAldrich, China) was injected into the mice twice with an interval of 3 weeks. The mouse plasma was collected 10 days after the last injection for inhibitor determination.

### Mouse blood samples

Blood was collected through the retro-orbital sinususing 3.8% sodium citrate as the anticoagulant. Blood samples were counted using an animal blood counter (Mindray, China). Plasma, platelets and platelet lysates were prepared as previously described^16^. Three mixtures of platelet agonists were used for platelet activation. The CAE agonist mixture (1 mM CaCl_2_,20 μM ADP and 250 μM epinephrine[KingYork, China]), the CAEP agonist mixture (1 mM CaCl_2_,20 μM ADP, 250 μM epinephrine, and 250 μM proteaseactivated receptor 4 agonist peptide GYPGKF [NJPeptide, China]), and the exposure agonist mixture (10 mM CaCl_2_, 40 μM ADP, 500 μM epinephrine and 250 μM GYPGKF peptide [Chinese Peptide, China]) were used in the alcohol exposure experiment.

### FXa assay

FXa in platelets was measured by a human FX specific enzyme-linked immunosorbent assay (ELISA) as reported^10^. Briefly, a goat anti-human FX polyclonal antibody (Affinity Biologicals, Ancaster, Canada) was used as a capture antibody, and an HRP-conjugated goat anti-human FX polyclonal antibody (Affinity Biologicals) was used as a detecting antibody. The Ultra TMB-ELISA substrate (Thermo Scientific, China) was used for detection. Recombinant FXa (Haematologic Technologies, USA) was used as a standard.

### FIX inhibitor assay

FIX inhibitors in mouse plasma were determined with the modified Bethesda assay as previously described^16^. An FIX activity assay kit (HYPHEN BioMed, France) was used to measure FIX activity following the manufacturer’s instruction.

### Tail bleeding assay

The tail bleeding assay was designed based on previous work^16^. The mouse was placed on a warm pad after anesthetized by 2.5% avertin. The tail was preheated by immersing it into 37 °C saline for 2 min and then transected at the diameter of 1.5 mm and immediately immersed into a conical tube containing 12 ml saline at 37 °C for 1h. The volume of blood loss was evaluated by measuring hemoglobin after lysis of red blood cells.

### Thrombin-antithrombin III complex (TAT), fibrinogen and d-Dimer assays

Mouse plasma was used for the measurement of TAT, fibrinogen and d-Dimer as previously reported^16^.

### Flow cytometry analysis

Isolated or activated platelets were resuspended in Tyrode’s buffer and stained with a PE conjugated P-selectin antibody (Thermo) in a dark room for 1h and fixed in1% paraformaldehyde for flow cytometry analysis.

### Alcohol exposure

We prepared 10% and 35% ethanol solution from absolute ethanol (99–100%). 10% and 35% ethanol were given to the mice through oral gavage. The dosing volume was 15% of the blood volume of a mouse, which was calculated as 8% of the body weight. Therefore, the dose of ethanol is 0.96 g/kg and 3.36 g/kg for 10% and 35%, respectively.

### Confocal analysis

Confocal analysis of FXa in platelets was performed as previously described^16^. Briefly, resuspended platelets at the concentration of 20 × 10^6^ platelets/ml were cytospinned onto slides, fixed, permeabilized, and stained with a goat anti-human FX antibody (Affinity Biologicals) as the primary antibody, an AlexaFluor 488-conjugated donkey anti-goat IgG antibody (Invitrogen, USA) as the secondary antibody.

### Statistical analysis

All results were presented by mean ± SD. We used a two-tailed Student’s t-test to compare two groups, used a one-way analysis of variance (ANOVA) and a Tukey’s multiple comparisons test or Kruskal–Wallis nonparametric test and Dunn's multiple comparisons test to compare three or more groups. *P* < 0.05 was considered significant. Studies were neither randomized nor blinded, as all animal experiments were performed with homogeneous age, strain and similar variance.

## Results

### FXa could be stored stably in 2bFXa-HB mouse platelets and was functional

**Figure 1:**
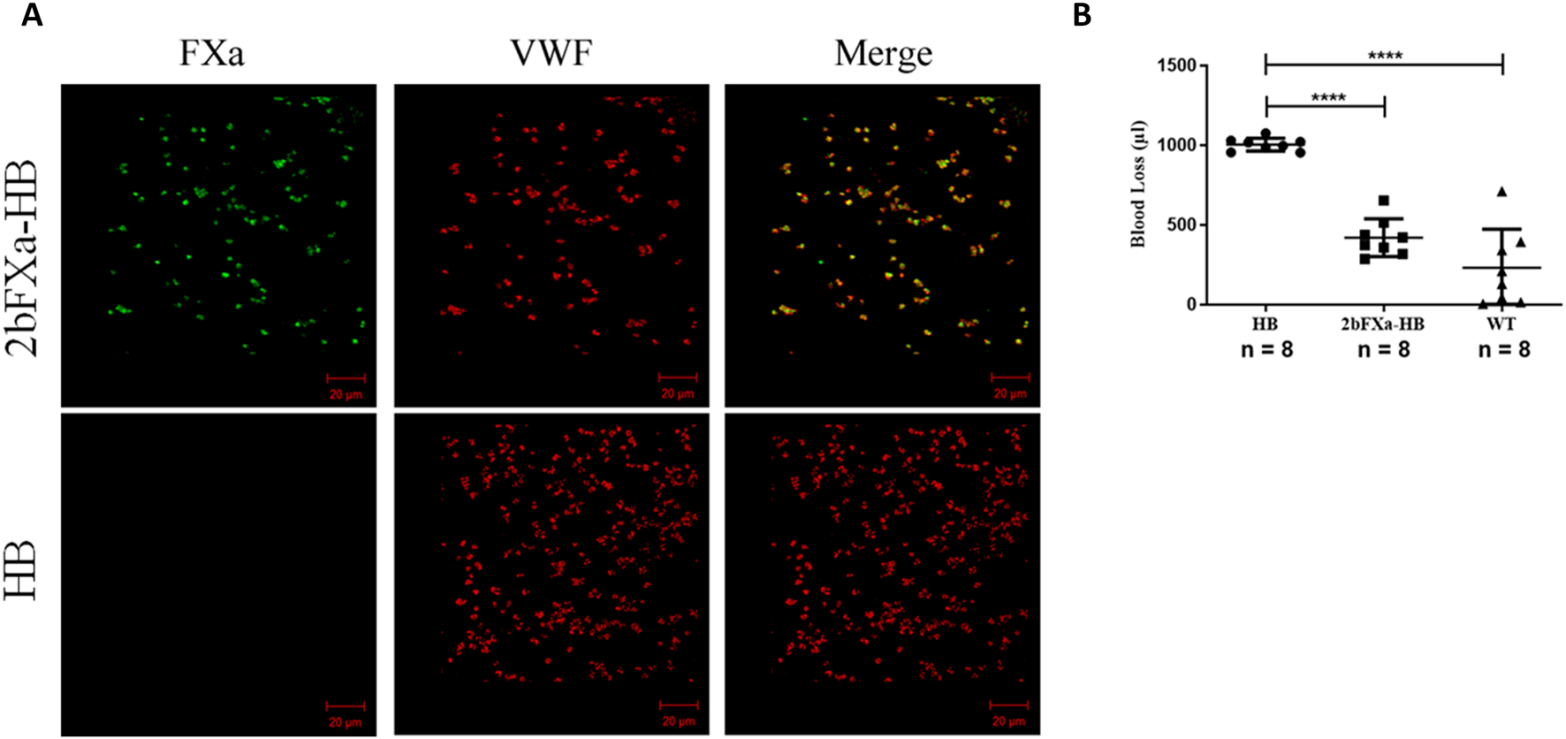
FXa stored in platelets of 2bFXa-HB mice and functional. **(A)** Representative confocal images of FXa in platelets. Platelets from 2bFXa-HB mice (top panel) and HB mice (bottom panel) were immunefluorescently stained for FXa and VWF. FXa was shown in the platelets of 2bFXa-HB mice and colocalized with VWF. VWF, von Willebrand factor. Scale bar = 20 µm. **(B)** Tail bleeding assay of the HB, 2bFXa-HB and wildtype (WT) mice. Data were analysed by one-way ANOVA with Tukey's multiple comparisons test. The numbers of samples are indicated. ****P < 0.0001.

We crossbred 2bFXa transgenic mice with HB mice, and through multiple generations of breeding and rigorous screening, we successfully established a stable 2bFXa-HB transgenic mouse line characterized by the consistent expression of FXa within platelets. Our analysis using confocal microscopy is consistent with colocalization of FXa with Von Willebrand factor (VWF) in the platelets of these transgenic mice (Fig. 1A), as we observed after gene therapy^10^.

To assess the functional impact, we conducted a mouse tail bleeding assay. The results demonstrated a significantly reduced blood loss volume in the 2bFXa-HB mice compared to the HB mice. Importantly, this reduction was not statistically different from the blood loss observed in the wildtype (WT) mice (Fig. 1B). These findings strongly suggest that platelet-derived FXa effectively improves the bleeding phenotype, validating its functionality in this context.

### Levels of thrombotic risk markers were not elevated in 2bFXa-HB mice

The enhanced activation of platelets is commonly associated with an increased risk of thrombosis. Since FXa is not naturally present in platelets under normal conditions, it is crucial to investigate whether the ectopic expression of FXa influences platelet activation. To address this, we employed two different mixtures of platelet agonists to activate platelets, with P-selectin serving as a marker of platelet activation.

**Figure 2:**
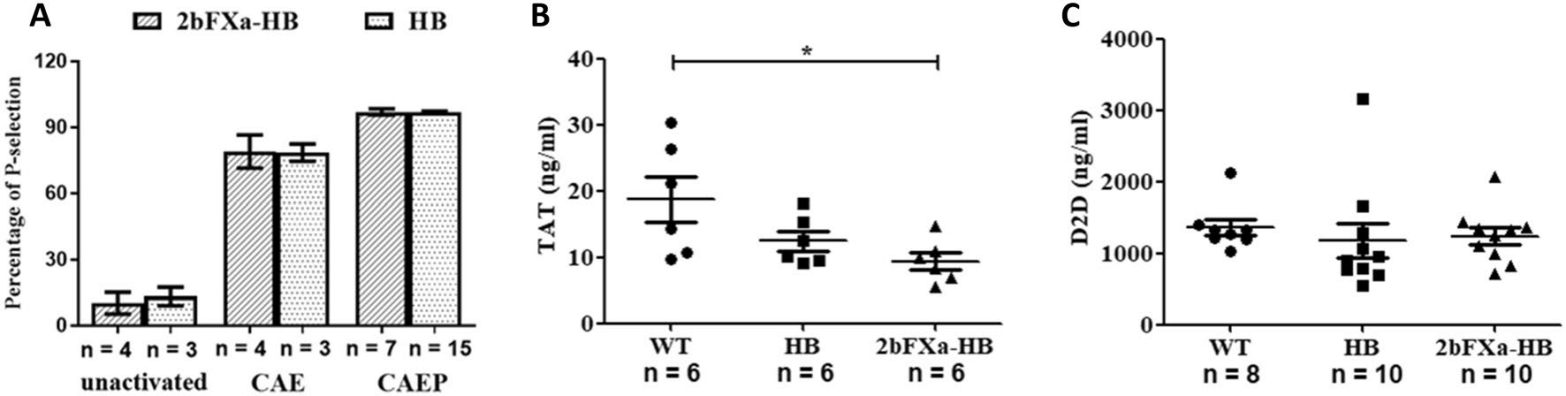
The activation of platelets and thrombotic risk markers did not changed in 2bFXa-HB mice. (**A)** The percentage of P-selectin positive platelets under different condition. unactivated: platelets were not activated; CAE: platelets were treated with CAE agonist mixture; CAEP: platelets were treated with CAEP agonist mixture. Data in 2bFXa-HB were compared with the corresponding data in HB by the Student’s t-test. No significant differences were found. **(B)** The amount of thrombin-antithrombin complex (TAT) in plasma of WT, HB and 2bFXa-HB mice. Data were analysed by one-way ANOVA with Tukey's multiple comparisons test. **(C)** The amount of D-Dimer (D2D) in plasma of WT, HB and 2bFXa-HB mice. Data were analysed by Kruskal–Wallis test with Dunn's multiple comparisons test. No significant differences were found. The numbers of samples are indicated. *P < 0.05.

After isolating and activating platelets from both 2bFXa-HB and HB mice using the CAE or CAEP agonist mixture, we observed no significant difference in the percentage of activated platelets between the two groups under either condition (Fig. 2A). This suggests that platelets from 2bFXa-HB mice exhibit a comparable response to agonist stimulation when compared to platelets from HB mice.

When it comes to assessing thrombotic risk, elevated levels of TAT and D-Dimer are typically relevant indicators. Our results indicate that the TAT level in 2bFXa-HB mice is similar to that in HB mice and significantly lower than that in WT mice (Fig. 2B). Additionally, there were no significant differences in D-Dimer levels when comparing 2bFXa-HB mice to WT and HB mice (Fig. 2C).

### Platelet-derived FXa corrected the bleeding phenotype of HB in presence of FIX inhibitors

The development of FIX inhibitors in a patient's plasma after replacement therapy poses a significant challenge for treatment. To address this issue, we immunized the 2bFXa-HB mice with recombinant human FIX to induce anti-FIX inhibitors in the mice. Subsequently, we conducted a tail bleeding assay to assess the impact.

**Figure 3:**
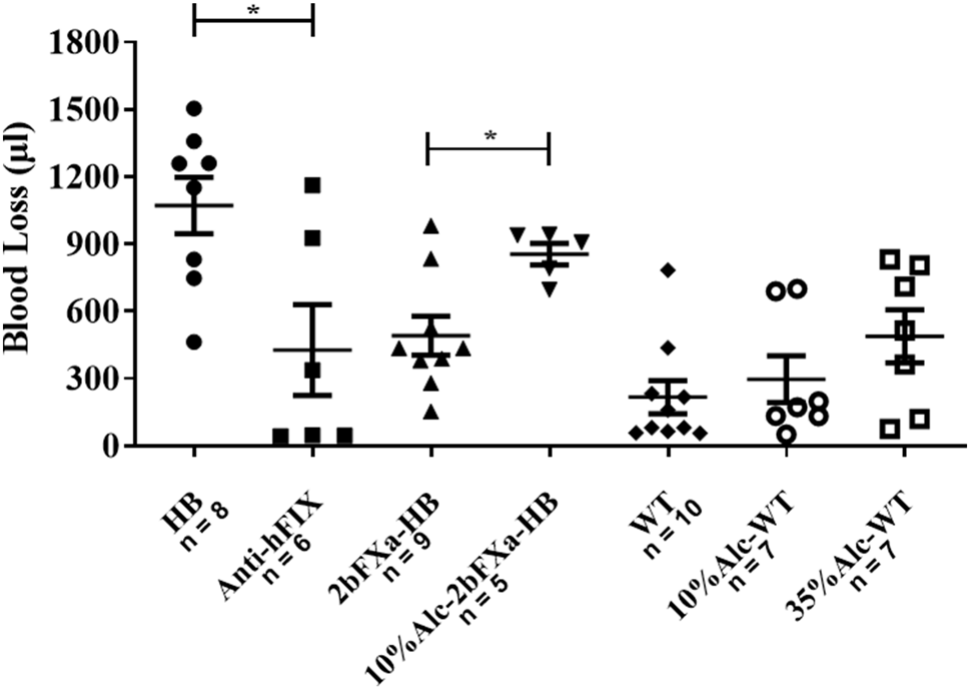
Tail bleeding assay of 2bFXa-HB mice under different conditions. The blood loss volume of each group was shown. Anti-hFIX: 2bFXa-HB mice developed anti-hFIX inhibitors; 10% Alc-2bFXa-HB: 2bFXa-HB mice after 10% alcohol exposure; 10% Alc-WT: wildtype mice after 10% alcohol exposure; 35% Alc-WT: wildtype mice after 35% alcohol exposure. A Student’s T-test was performed for comparing HB and Anti-hFIX groups, as well as 2bFXa-HB and 10%Alc-2bFXa-HB groups. A Kruskal–Wallis test with Dunn's multiple comparisons test was performed for analyzing three WT groups, no significant differences were found. The numbers of samples are indicated. *P < 0.05.

The mean inhibitor titer ranged from 27 BU/ml to 142 BU/ml, with an average of 77.3 ± 40.4 BU/ml. As expected, the blood loss in 2bFXa-HB mice containing inhibitors was similar to that in 2bFXa-HB mice and significantly lower than that in HB mice (Fig. 3). This demonstrates that platelet-derived FXa is still capable of rescuing coagulation function even in the presence of high-titer anti-FIX inhibitors.

### Acute alcohol exposure of 2bFXa-HB mice

**Figure 4:**
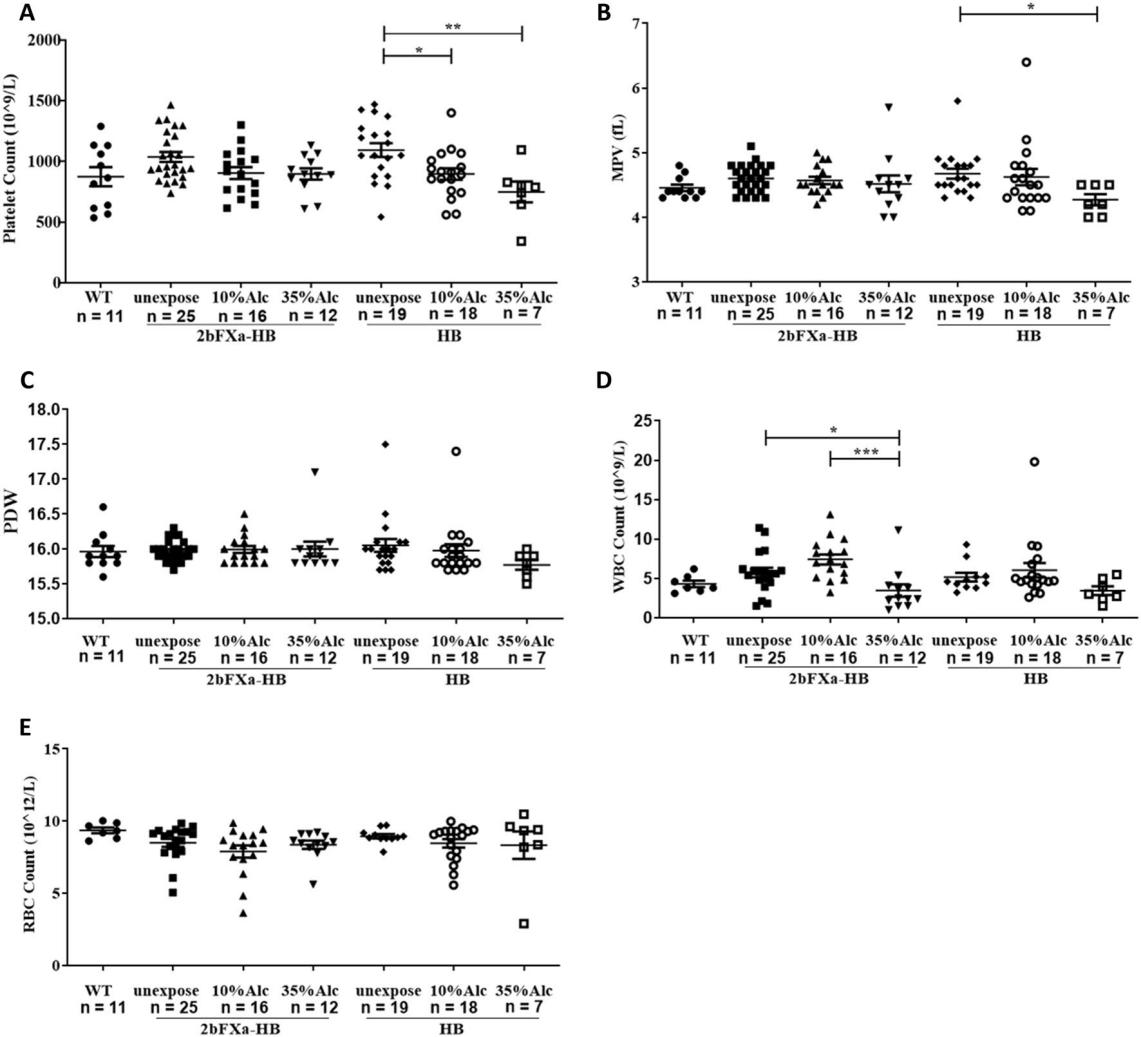
Counting of whole blood cells after alcohol exposure. The count of platelets **(A)**, mean platelet volume (MPV) **(B)**, platelet distribution width (PDW) **(C)**, white blood cell count (WBC) **(D)** and red blood cell count (RBC) **(E) **in mice were shown. unexpose: mice without alcohol exposure; 10% Alc: mice with 10% alcohol exposure; 35% Alc: mice with 35% alcohol exposure. A Kruskal–Wallis test with Dunn's multiple comparisons test was performed for analyzing the groups in 2bFXa-HB and the groups in HB, except for the HB groups in **(A),** which used one-way ANOVA with Tukey's multiple comparisons test. The groups in 2bFXa-HB were compared to the conresponding groups in HB by a Student’s t-test, no significant differences were found. The unexposed groups in 2bFXa-HB and HB mice were compared with WT mice by a Kruskal-Wallis test with Dunn's multiple comparisons test, no significant differences were found. The numbers of samples are indicated. *P < 0.05, **P < 0.01, ***P < 0.001.

Alcohol has a significant impact on platelets and the coagulation system. We examined whether alcohol exposure would influence FXa-containing platelets. Mice were given 10% or 35% alcohol once, and 1.5 h later, mouse blood was collected for analysis. The platelet count in HB mice after administration of 10% alcohol or 35% alcohol was significantly lower than that in the unexposed group. There was no significant difference between the 10% and 35% groups. However, the dose–effect of decreasing platelet count with increasing alcohol exposure concentration was apparent. There were no significant differences among the 2bFXa-HB groups, although there was a clear reduction in platelet count in the 10% and 35% groups compared to the unexposed group. There were no significant differences between the corresponding groups in 2bFXa-HB and HB mice (Fig. 4A).

The mean platelet volume (MPV) of HB mice after 35% alcohol exposure was significantly lower than that of the unexposed group. No significant difference was found between the 10% group and the unexposed group. For 2bFXa-HB mice, there were no significant differences between the exposed and unexposed groups, and also no significant difference between the corresponding groups of 2bFXa-HB and HB mice (Fig. 4B). No significant differences were found among the groups of platelet distribution width (PDW) (Fig. 4C). Taken together, alcohol exposure substantially reduced platelet count of both 2bFXa-HB and HB mice, the effect was dose-dependent.

White blood cell (WBC) count of the 35% exposure group of 2bFXa-HB mice was significantly lower than that of the unexposed and 10% exposure groups. There was no significant difference between the latter two groups. In HB mice, the WBC count of the 35% exposure group was also lower than that of the unexposed and 10% exposure groups, but there was no significant difference (Fig. 4D). Meanwhile, no significant difference was also found between the corresponding groups of 2bFXa-HB and HB mice. For the red blood cell (RBC) count, there were no significant differences among all groups (Fig. 4E). In summary, these results indicate that acute alcohol exposure affected platelet countand WBC count, but no significant difference was found between 2bFXa-HB and HB mice in response to the exposure, suggesting that FXa storing in platelets does not interfere with the response of platelets to alcohol.

### The influence of acute alcohol exposure on functions of platelets and blood coagulation system

**Figure 5:**
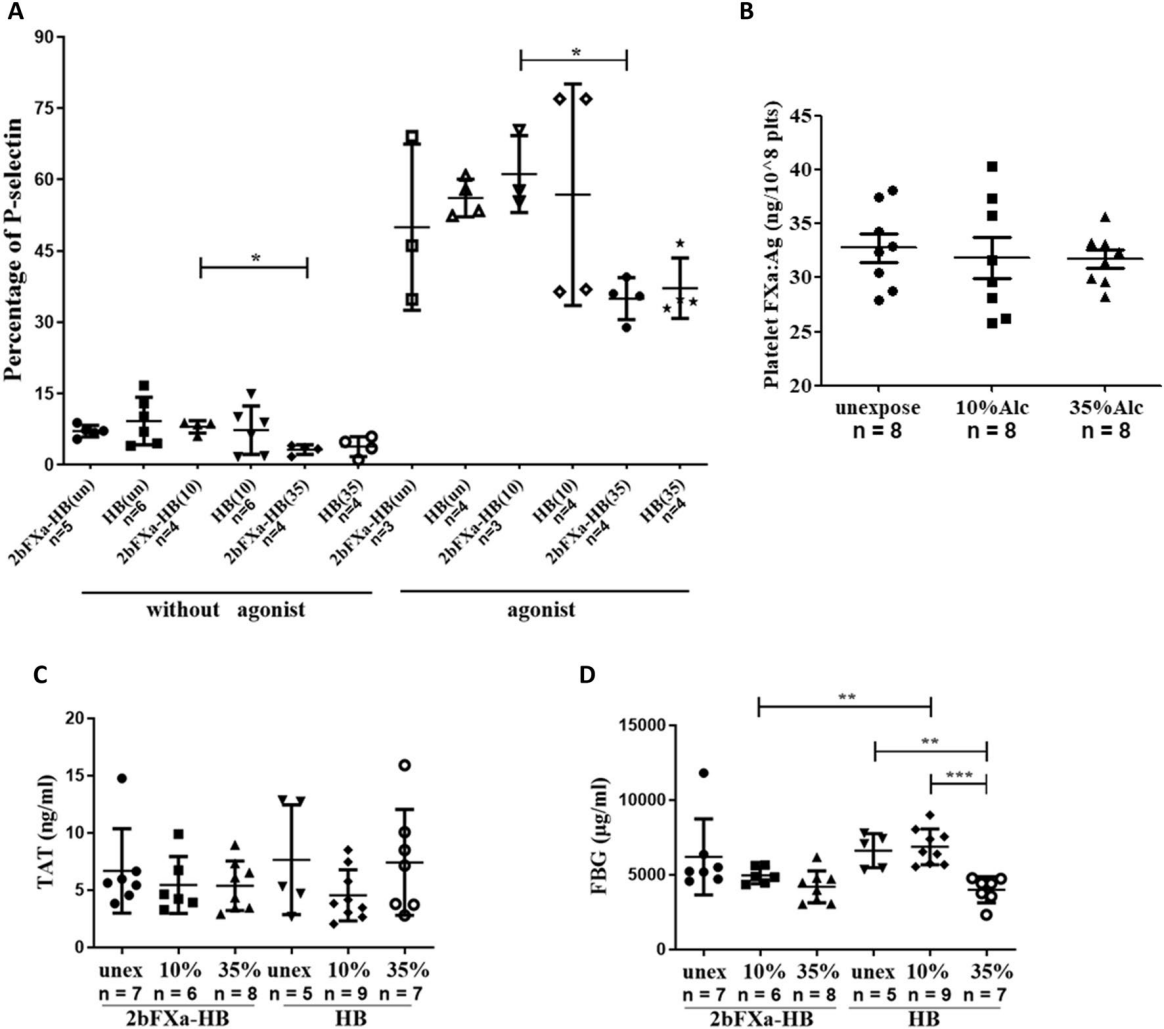
The influence of alcohol exposure on platelets. **(A)** Percentage of P-selectin positive platelets with or without activation. The agonist use for platelet activation was the exposure agonist mixture. A one-way ANOVA with Tukey's multiple comparisons test was used for analyzing HB groups without agonist and 2bFXa-HB groups with agonist; a Kruskal–Wallis test with Dunn's multiple comparisons test was performed for analyzing 2bFXa-HB groups without agonist and HB groups with agonist. A Student’s test was performed to compare the groups in 2bFXa-HB with the corresponding groups in HB, no significant differences were found. **(B)** FXa antigen level (FXa:Ag) in 2bFXa-HB mice after alcohol exposure. Data were analyzed by a one-way ANOVA with Tukey's multiple comparisons test, no significant differences were found. **(C)** The concentration of TAT complex in 2bFXa-HB and HB mice. The 2bFXa-HB groups were analyzed by a Kruskal–Wallis test with Dunn's multiple comparisons test. The HB groups were analyzed by a one-way ANOVA with Tukey's multiple comparisons test. The groups in 2bFXa-HB were compared with the corresponding groups in HB by a Student’s t-test. No significant differences were found. **(D)** The concentration of fibrinogen (FBG) in 2bFXa-HB and HB mice. The 2bFXa-HB groups were analyzed by a Kruskal–Wallis test with Dunn's multiple comparisons test. The HB groups were analyzed by a one-way ANOVA with Tukey's multiple comparisons test. The groups in 2bFXa-HB were compared with the corresponding groups in HB by a Student’s t-test. Un, unex or unexpose: unexposed to alcohol; 10, 10% Alc or 10%: 10% alcohol exposure; 35, 35% Alc or 35%: 35% alcohol exposure. The numbers of samples are indicated. *P < 0.05, **P < 0.01, ***P < 0.001.

To further investigate the effects of alcohol exposure on FXa-storing platelets, we isolated platelets from mice subjected to alcohol exposure and examined their platelet activation capacity. As shown in Fig. 5A, P-selectin-positive platelets in the alcohol-exposed groups exhibited a significant increase in response to platelet activation, similar to the unexposed group.

In both their inactive and activated states, mice from both the 2bFXa-HB and HB groups exposed to 35% alcohol displayed lower numbers of P-selectin-positive cells compared to their respective unexposed and 10% exposure groups. However, the statistically significant difference was observed only between the 35% group and the 10% group for both inactive and activated 2bFXa-HB mice. Furthermore, the percentage of P-selectin-positive platelets in the 10% exposure group for both 2bFXa-HB and HB mice closely resembled that of their respective unexposed groups (Fig. 5A).

No discernible differences were found between the corresponding groups of 2bFXa-HB and HB mice, irrespective of whether the platelets were exposed to 10% or 35% alcohol or whether the platelets were activated (Fig. 5A). These results suggest that while 35% alcohol exposure inhibits platelet activation in both 2bFXa-HB and HB mice, 10% alcohol exposure does not. Additionally, the presence of stored FXa in HB platelets does not alter platelet activation properties.

The platelet FIX levels did not exhibit significant differences among the unexposed, 10%, and 35% exposure groups (Fig. 5B), indicating that FXa remains stably stored in platelets even under conditions of alcohol exposure. Similarly, there were no significant variations in TAT levels across all groups (Fig. 5C).

Fibrinogen levels were notably reduced in both 2bFXa-HB and HB mice following exposure to 35% alcohol, compared to their respective unexposed groups. However, significant differences were only observed in HB mice. Within the 2bFXa-HB groups, there was a dose-dependent reduction in fibrinogen levels with increasing alcohol concentration. Notably, the fibrinogen level in the 10% exposure group of HB mice closely resembled that of the unexposed group, and it was significantly higher than that in the 35% exposure group of HB mice and the 10% exposure group of 2bFXa-HB mice (Fig. 5D).

These findings collectively indicate that there is no evidence of an increased thrombotic risk in 2bFXa-HB mice following alcohol exposure.

In the tail bleeding assay, it was evident that the blood loss in 10% ethanol-exposed 2bFXa-HB mice was significantly higher than that in unexposed 2bFXa-HB mice, approximately doubling the loss observed in unexposed 2bFXa-HB mice and approaching the levels seen in HB mice. This difference was sufficiently pronounced to underscore the impact of alcohol, rendering further experimentation with 35% ethanol unnecessary. Notably, we observed a noticeable trend of increased blood loss in WT mice following exposure to both 10% and 35% alcohol, although no statistically significant differences emerged among the WT groups (Fig. 3).

These findings collectively suggest that alcohol exposure adversely affected the coagulation system of the mice, resulting in an attenuated hemostatic effect of FXa-storing platelets in 2bFXa-HB mice.

## Discussion

Over the past few decades, the targeted expression of human FVIII in platelets has demonstrated therapeutic efficacy in mouse^17,18^, rat^19^, and dog^20^ models of HA, without encountering issues of immunogenicity or thrombotic risks^21,22^. Additionally, our previous work showed that targeting the expression of FIX Padua in platelets proved therapeutically effective in FIX-deficient mice, with no observed thrombotic risks^16^. While platelet-targeted expression of FIX can alleviate the coagulation disorder phenotype in HB mice, its effectiveness is significantly diminished in the presence of FIX inhibitors^23^. To address this challenge, we introduced the concept of targeting FXa expression in platelets and demonstrated the therapeutic potential of platelet-derived FXa in mouse models of both HA and HB, particularly in the presence of FIX inhibitors^10^. In the present study, we aimed to assess the efficacy and safety of platelet-derived FXa in a transgenic HB mouse model. Concurrently, we investigated the effects of acute alcohol exposure on platelets and coagulation function.

In this study, we have demonstrated that platelet-derived FXa effectively ameliorates the bleeding phenotype in HB mice, even in the presence of FIX inhibitors. Furthermore, the levels of TAT, d-Dimer, and fibrinogen in 2bFXa-HB mice were not elevated when compared to those in HB mice. These results are consistent with our prior research^10^ and provide support for the hypothesis that platelet-stored FXa does not increase the risk of thrombosis. Nevertheless, it is important to note that the current findings may not be sufficient to conclusively establish this point. Further investigations using different thrombotic models and tests are warranted to fully address this aspect.

An intriguing observation was the variance in d-Dimer levels between this study and our previous work^16^. This variance could potentially be attributed to differences in blood collection methods. In this study, blood was obtained from the orbital sinus, while in our earlier research, blood was collected via cardiac puncture. It has been documented that blood collected from the orbital sinus is more prone to activation than blood collected through cardiac puncture^24^. This underscores the sensitivity of d-Dimer as a marker to variations in blood collection methods.

With regard to platelets, our investigations revealed no discernible differences in the activation capacity of FXa-containing platelets when comparing 2bFXa-HB mice to HB mice. Similarly, no disparities were noted in whole blood cell counts when comparing 2bFXa-HB mice to HB and WT mice. Moreover, MPV and PDW remained unchanged in 2bFXa-HB mice. Collectively, these results indicate that targeting FXa expression in platelets does not interfere with the physiological functions of platelets. Throughout the course of this study, we maintained the transgenic mice for over 40 generations, and we did not observe any abnormal phenotypes or health issues associated with thrombogenesis. Furthermore, there was no increase in mortality when comparing these transgenic mice to HB mice.

Alcohol is a widely abused substance with a substantial impact on platelet function. Previous research has shown that alcohol can lead to decreased platelet count, hinder platelet aggregation, and induce platelet apoptosis^25^. As platelet-targeted gene therapy for hemophilia progresses toward clinical trials, it becomes imperative to elucidate the effects of alcohol on platelet function, particularly when coagulation factors are stored within them. In this context, we employed a platelet-FXa transgenic mouse model to investigate how alcohol influences FXa-stored platelets and the hemostatic system.

In our study, we chose to use 10% and 35% alcohol solutions to simulate the consumption of beer/wine and liquor, respectively, and designed an acute exposure model to mimic binge drinking scenarios. Our findings revealed a substantial reduction in platelet count in both 2bFXa-HB and HB mice following acute exposure to 10% and 35% alcohol, with a clear dose-dependent relationship between alcohol concentration and platelet count reduction. This outcome aligns with clinical observations that acute alcohol exposure can lead to thrombocytopenia^26^. Although the precise mechanism by which alcohol induces thrombocytopenia remains unclear, several explanations have been proposed. Some studies have suggested that alcohol exhibits direct toxicity to platelets or interferes with thrombopoiesis. Additionally, platelet apoptosis appears to accelerate after acute alcohol exposure. Furthermore, alcohol has been shown to induce alterations in platelet morphology^27,28^, and there is evidence that non-oxidative ethanol metabolites, such as fatty acid ethyl esters, can trigger platelet shape changes and the release of α-granules^29^. Therefore, we sought to determine whether acute alcohol exposure might trigger the release of FXa, potentially elevating the risk of thrombosis.

Our findings, however, indicate that the level of platelet-stored FXa in 2bFXa-HB mice remained unaltered following acute alcohol exposure. Additionally, there was no increase in plasma levels of TAT or fibrinogen, suggesting no signs of heightened thrombotic risk after alcohol exposure. Moreover, the MPV and PDW remained within the normal range in alcohol-exposed 2bFXa-HB mice, indicating that alcohol did not induce changes in platelet shape during exposure. Intriguingly, while MPV significantly decreased in HB mice exposed to 35% alcohol compared to their unexposed counterparts, which contrasts with previous findings in chronic alcohol consumption patients^28^, there were no such differences observed between the corresponding exposure groups for 2bFXa-HB and HB mice.

These results collectively suggest that acute alcohol exposure leads to a reduction in platelet count, but it does not appear to affect FXa storage or induce thrombotic risk in 2bFXa-HB mice. Furthermore, the observed changes in MPV do not align with those seen in chronic alcohol consumption.

Conversely, existing studies have indicated that alcohol not only hampers platelet activation and aggregation^27,30,31^ but also exerts an influence on the coagulation system^30^. In our investigation, we observed a diminished proportion of activated platelets in the 35% alcohol exposure group, affecting both 2bFXa-HB and HB mice, with a notably significant reduction noted in 2bFXa-HB mice. These results suggest a dampening effect on platelet activation capacity during acute exposure to 35% alcohol. Furthermore, there was a conspicuous reduction in fibrinogen levels observed in the 35% alcohol exposure group, which aligns with prior observations^32,33^. However, we did not detect a significant decline in fibrinogen levels in the 10% alcohol exposure groups. Nevertheless, there remained a noticeable trend towards reduced fibrinogen levels following alcohol exposure in these groups.

In the tail bleeding assay, the 10% alcohol exposure group of 2bFXa-HB mice exhibited significantly higher blood loss than the unexposed group, approaching the levels observed in HB mice. This outcome can be attributed to a combination of factors, including the reduction in platelet count following alcohol exposure and the inhibitory effects of alcohol on the coagulation system. We noted decreases in both platelet count and fibrinogen levels in the 10% alcohol-administered 2bFXa-HB mice, which may have contributed to the increased blood loss, although the data did not reach statistical significance. Nevertheless, the reduction in platelet count in alcohol-administered HB mice was significant, providing evidence of alcohol's impact on platelets.

Furthermore, we observed an escalation in blood loss with increasing alcohol consumption in WT mice, highlighting the dose-dependent effect of alcohol on impairing the coagulation process in mice. Notably, the adverse effects of acute alcohol exposure were more pronounced in 2bFXa-HB mice compared to WT mice. This is expected, as 2bFXa-HB mice heavily rely on platelet-derived FXa for hemostasis, while WT mice possess a normal coagulation system to maintain hemostatic activities.

In summary, our results indicate that even mild alcohol exposure can lead to platelet reduction, potentially influencing the blood clotting process. This effect becomes more pronounced in recipients of platelet-targeted gene therapy. Notably, significant suppression of platelet activation and a reduction in fibrinogen were observed at higher alcohol concentrations. In such cases, the combination of thrombocytopenia, inhibited platelet activation, and reduced fibrinogen levels could further hinder the coagulation process. Additionally, impairments in the coagulation system beyond platelets and fibrinogen may also contribute to these consequences.

Overall, our study demonstrates that platelet-derived FXa is effective and safe for HB, even in the presence of high-titer anti-FIX inhibitors. It provides direct evidence that alcohol consumption can inhibit the therapeutic effect of platelet-derived FXa but may not increase thrombotic risk. It is reasonable to extrapolate that the conclusions drawn in this study also apply to hemophilia gene therapy strategies targeting FVIII or FIX expression in platelets. Therefore, controlling alcohol consumption, especially binge drinking, should be considered for hemophilia patients undergoing platelet-targeted gene therapy.

## Data Availability

All data generated or analysed during this study are included in this published article.
